# Supplementary material for: Methylated Cell-Free Tumor DNA in Sputum as a Tool for Diagnosing Lung Cancer—A Systematic Review and Meta-Analysis
Source: Cancers (Basel). 2024 Jan 24;16(3):506. doi: 10.3390/cancers16030506 (PMC10854681; doi:10.3390/cancers16030506)
Supplement: Supplementary file 1 [file cancers-16-00506-s001.zip › cancers-2827974-supplementary.pdf]

Supplementary Materials

Methylated Cell-Free Tumor DNA in Sputum as a Tool for Diagnosing Lung Cancer—A Systematic Review and Meta-Analysis

Sara Witting Christensen Wen [1,2,*,†], Morten Borg [3,†], Signe Timm [1,2], Torben Frøstrup Hansen [1,2], Ole Hilberg [2,3] and Rikke Fredslund Andersen [4]

[1] Department of Oncology, Vejle Hospital, University Hospital of Southern Denmark, 7100 Vejle, Denmark
[2] Department of Regional Health Research, University of Southern Denmark, 5000 Odense, Denmark
[3] Department of Medicine, Vejle Hospital, University Hospital of Southern Denmark, 7100 Vejle, Denmark
[4] Department of Biochemistry and Immunology, Vejle Hospital, University Hospital of Southern Denmark, 7100 Vejle, Denmark
* Correspondence: sara.witting.christensen.wen@rsyd.dk
† These authors contributed equally to this work.

Search strings

All searches were carried out as described in the Methods-section. The three blocks were combined with the Boolean operator 'AND' to retrieve the relevant results.

EMBASE and EMBASE Classic 21-08-2023

Block 1: LUNG CANCER
exp lung cancer/
((lung or pulmonary or bronchial or bronchiogenic or bronchogenic or bronchiolo alveolar or bronchial or non small cell or small cell) adj3 (cancer* or neoplasm* or maligna* or carcinoma* or adenocarcinoma* or squamous cell carcinoma)) or (schneeberg adj3 disease)

Block 2: SPUTUM
exp sputum/ or exp sputum examination/ or exp sputum analysis/ or exp sputum cytodiagnosis/ or exp sputum smear/ exp bronchus mucus/ or exp trachea mucus/ or exp mucus/
sputum or expectorat* or phlegm or (sputum adj3 induc*) or (spontaneous adj3 sputum) or (forced adj3 sputum) or saliva or spit or mucus

Block 3: METHYLATED TUMOR DNA
exp circulating tumor DNA/ or exp liquid biopsy/
((tumor or tumour or cell free or circulating or methylat* or tumor specific or tumour specific) adj3 (dna or gene)) or ctdna or cfdna or liquid biops*

Ovid Medline All (blocks 1 and 3 identical with Embase) 21-08-23

Block 2: SPUTUM
exp Sputum/ or exp Mucus/
sputum or expectorat* or phlegm or (sputum adj3 induc*) or (spontaneous adj3 sputum) or (forced adj3 sputum) or saliva or spit or mucus

Web of Science 21-08-23

Block 1: LUNG CANCER
((lung or pulmonary or bronchial or bronchiogenic or bronchogenic or “bronchiolo alveolar” or bronchial or “non small cell” or “small cell”) NEAR/2 (cancer* or neoplasm* or maligna* or carcinoma* or adenocarcinoma* or “squamous cell carcinoma”)) or (schneeberg NEAR/2 disease)

Block 2: SPUTUM
sputum or expectorat* or phlegm or (sputum NEAR/2 induc*) or (spontaneous NEAR/2 sputum) or (forced NEAR/2 sputum) or saliva or spit or mucus

Block 3: CIRCULATING TUMOR DNA
(tumor DNA) or (tumour DNA) or (cell free DNA) or (circulating DNA) or (methylat* DNA) or (tumor specific DNA) or (tumour specific DNA) or ctDNA or cfDNA or (liquid biops*)

Cochrane Library 21-08-23

Block 1: LUNG CANCER
MeSH: lung neoplasms
((lung or pulmonary or bronchial or bronchiogenic or bronchogenic or bronchiolo NEXT alveolar or bronchial or non NEXT small NEXT cell or small NEXT cell) NEAR/2 (cancer* or neoplasm* or maligna* or carcinoma* or adenocarcinoma* or squamous NEXT cell NEXT carcinoma)) or (schneeberg NEAR/2 disease)

Block 2: SPUTUM
MeSH: sputum
MeSH: mucus
sputum or expectorat* or phlegm or (sputum NEXT induction) or (induced NEXT sputum) or (spontaneous NEXT sputum) or (forced NEXT sputum) or (sputum NEXT analysis) or (sputum NEXT examination) or (sputum NEXT smear) or saliva or spit or mucus or (tracheal NEXT mucus) or (bronchial NEXT mucus)

Block 3: CIRCULATING TUMOR DNA
MeSH: circulating tumor DNA or liquid biopsy or DNA methylation
((tumor or tumour or (cell NEXT free) or circulating or methylat* or (tumor NEXT specific) or (tumour NEXT specific)) NEAR/2 (dna or gene)) or ctdna or cfdna or (liquid NEXT biops*)

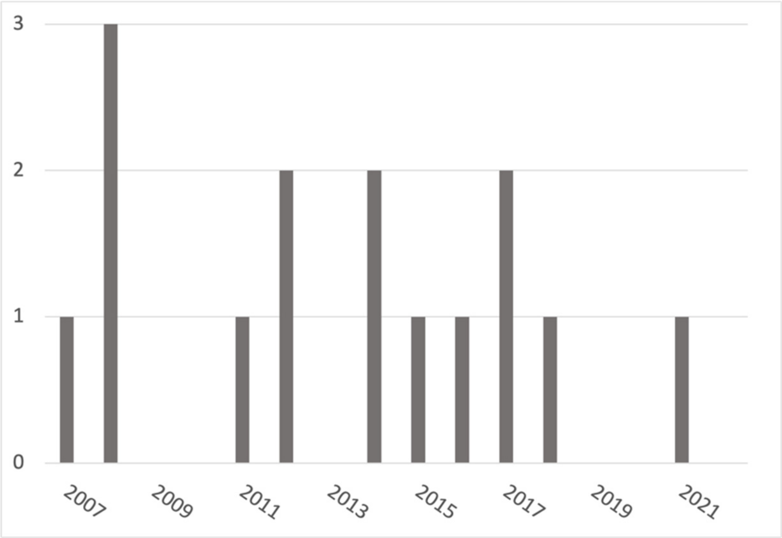

Figure S1. Number of studies published by year.

Table S1. Sample type, collection method, and DNA extraction. Spontaneous sputum: Sputum produced spontaneously and unaided. Induced sputum: Sputum produced after inhalation of a nebulized, hypertonic saline solution. Lung Flute: Sputum produced after using the Lung Flute device.

Study ID	Sample type	Collection	Part of the biological specimen used?	DNA extraction kit	DNA amount used for analysis	DNA quality check
Shivapurkar, 2007 [31]	Spontaneous sputum	Collected from multiple days and pooled	Not described	QIAamp DNA blood mini kit (QIAgen, Venlo, the Netherlands)	1 ml bisulfite-converted genomic DNA (corresponds to approx. 50 ng initial DNA)	Not reported
Shivapurkar, 2008 A [32]	Spontaneous sputum	Collected from multiple days and pooled	Not described	QIAamp DNA blood mini kit (QIAgen, Venlo, the Netherlands)	Not reported	Not reported
van der Drift, 2008 [33]	Spontaneous sputum	Collected from multiple days and pooled	Pellet and supernatant compared	QIAamp DNA Blood Maxi kit (Qiagen) according to the ''Blood and Body Fluid Spin Protocol''. The same method was used for supernatant and cells.	The amount of modified DNA used in the first PCR step was up to 400 ng for the DNA from sputum cell pellets and 0.04—319 ng for DNA from sputum supernatants (the maximal amount of the DNA material available)	Not reported
Shivapurkar, 2008 B [34]	Spontaneous sputum	Collected from multiple days and pooled	Not described	QIAamp DNA blood mini kit (QIAgen, Venlo, the Netherlands)	Reference Shivapurkar, 2007 [31]	Not reported
Hwang, 2011 [35]	Induced sputum	Not described	Not described	DNA was extracted from tissue samples using a DNeasy tissue kit (Qiagen Gmbh, Hilden, Germany) - nothing mentioned specifically for sputum, but presumed to be the same.	2 µL of bisulfite modified DNA (extracted DNA was adjusted to 40 ng/µL, 2 µg of the sample was bisulfite treated, and the converted DNA was eluted in 50 µL of the buffer)	DNA concentration was measured by spectrophotometry, quality check not reported
Hubers, 2012 [27]	Spontaneous sputum	Collected from multiple days and pooled	Not described	QIAamp Blood Mini Kit (Qiagen, Hilden, Germany)	200 ng of bisulphite-converted genomic DNA	PCR for the bisulphite-converted MYOD1 was used as quality control
Leng, 2012 [19]	Spontaneous sputum	Collected from multiple days and pooled	Pellet	DNA was isolated from sputum by protease digestion followed by phenol chloroform extraction and ethanol precipitation	100 to 150 ng of DNA was used for stage I PCR following modification with bisulfite	Not reported
Hubers, 2014 A [36]	Spontaneous sputum	Collected from multiple days and pooled	Not described	Not described	Not described	Not described
Hubers, 2014 B [29]	Spontaneous sputum	Collected from multiple days and pooled	Not described	Unsure - Only via references. Ref 8 in the paper mentions phenol-	Unsure. Ref 8 describes that 120-150 ng was used for stage 1 PCR.	Not reported

				chloroform, 12 references 13, and 13 uses QIAmp DNA mini kit.	Ref 13 corresponds to Shivapurkar, 2007 [31] and is described above.	
Hubers, 2015 [18]	Spontaneous sputum	Collected from multiple days and pooled	Not described	QIAamp Blood Mini Kit (Qiagen, Hilden, Germany) via ref Hubers 2012	200 ng of bisulphite-converted genomic DNA via ref Hubers 2012	PCR for the bisulphite-converted MYOD1 was used as quality control via ref Hubers 2012
Su, 2016 [20]	Induced sputum	Single-day sample	Not described	Unsure. QIAamp Blood Mini Kit (Qiagen, Hilden, Germany) via ref Hubers 2015, who reference Hubers 2012	Unsure. 200 ng of bisulphite-converted genomic DNA via ref Hubers 2015 who reference Hubers 2012	As described for Hubers 2015 and Hubers 2012
Hubers, 2017 [37]	Spontaneous sputum	Collected from multiple days and pooled	Not described	QIAamp Blood Mini Kit (Qiagen, Hilden, Germany) via ref Hubers 2012	200 ng of bisulphite-converted genomic DNA via ref Hubers 2012	As described for Hubers 2012
Hulbert, 2017 [28]	Spontaneous sputum	Collected from multiple days and pooled	Not described	Methylation on beads ("a process that allows DNA extraction and bisulfite conversion in a single tube via the use of silica super magnetic beads")	2 µL of bisulfite-converted DNA (but no concentration was mentioned)	β-actin was used as a control assay for the QMSP analysis
Su, 2018 [38]	Induced sputum	Single-day sample	Pellet	DNeasy kit (Qiagen, Valencia, CA)	2 µL of bisulfite-converted DNA (but no concentration was mentioned)	DNA quantified using Quantifiler Human DNA Quantification kit (Applied Biosystems, Foster City, CA)
Li, 2021 [30]	Other: Lung Flute	Single-day sample	Pellet	Qiagen DNeasy kit (Qiagen, Germantown, MD, USA)	1 µg DNA was eluted with 50 µL of elution buffer before bisulfite conversion, but the amount of DNA used for digital PCR is not described	Not described

Table S2. Comprehensive list of genes analyzed by the 15 studies included in the systematic review and meta-analysis. The frequency refers to the number of cohorts in which the gene was studied.

**Gene name**	**Frequency**
RASSF1A	13
APC	9
CYGB	8
3OST2	7
PRDM14	6
FAM19A4	4
HOXA9	4
PHACTR3	4
DAPK	3
SOX17	3
TAC1	3
Dal1	2
P16	2
PAX5B	2
CDO1	1
CXCL14	1
DLC1	1
FHIT	1
GATA	1
GATA5	1
HOXA7	1
Jph3	1
Kif1a	1
MAGE	1
MGMT	1
PAX5	1
PAX5A	1
PCDH20	1
SULF2	1
TCF21	1
ZFP42	1

Table S3. Sensitivity, specificity, and contingency data for the 15 studies included in the meta-analysis. CO, Colorado cohort; NM, New Mexico cohort.

Study ID	Gene name	Sensitivity	Specificity	Cases	Controls	Test positive Lung cancer positive	Test positive Lung cancer negative	Test negative Lung cancer positive	Test negative Lung cancer negative
Shivapurkar 2007	3OST2	30.8	100	13	25	4	0	9	25
	RASSF1A	38.5	100	13	25	5	0	8	25
	P16	23.1	100	13	25	3	0	10	25
	APC	23.1	100	13	25	3	0	10	25
Shivapurkar 2008 A	CYGB	30.8	100	13	25	4	0	9	25
vanderDrift 2008	RASSF1A supernatant	21.7	100	23	52	5	0	18	52
	RASSF1A pellet	42.9	98.5	28	68	12	1	16	67
Shivapurkar 2008 B	TCF21	54	100	13	25	7	0	6	25
Hwang 2011	HOXA9	70.7	55.1	76	109	54	49	22	60
Hubers 2012	RASSF1A cumulative	41.5	95.7	53	47	22	2	31	45
	APC cumulative	17	95.7	53	47	9	2	44	45
	CYGB cumulative	37.7	97.9	53	47	20	1	33	46
Leng 2012	MGMT CO	26.6	82.8	64	64	17	11	47	53
	DAPK CO	35.9	75	64	64	23	16	41	48
	PAX5B CO	42.2	73.4	64	64	27	17	37	47
	Dal1 CO	26.6	90.6	64	64	17	6	47	58
	PCDH20 CO	45.3	68.8	64	64	29	20	35	44
	Jph3 CO	29.7	82.8	64	64	19	11	45	53
	Kif1a CO	34.4	78.1	64	64	22	14	42	50
	DAPK NM	52.5	65.6	40	90	21	31	19	59
	PAX5B NM	40	74.4	40	90	16	23	24	67
	PAX5A NM	62.5	65.6	40	90	25	31	15	59
	Dal1 NM	35	83.3	40	90	14	15	26	75

	GATA5 NM	77.5	53.3	40	90	31	42	9	48
	SULF2 NM	77.5	44.8	40	90	31	50	9	40
	CXCL14 NM	22.5	94.4	40	90	9	5	31	85
Hubers 2014 A	RASSF1A	55	87	20	31	11	4	9	27
	APC	60	55	20	31	12	14	8	17
	CYGB	55	68	20	31	11	10	9	21
	3OST2	50	87	20	31	10	4	10	27
	PRDM14	65	81	20	31	13	6	7	25
	FAM19A4	75	26	20	31	15	23	5	8
	PHACTR3	60	68	20	31	12	10	8	21
Hubers 2014 B	RASSF1A set 1	41	96	98	90	40	4	58	86
	APC set 1	31	94	98	90	30	5	68	85
	CYGB set 1	40	87	98	90	39	12	59	78
	RASSF1A set 2	52	94	60	445	31	27	29	418
	APC set 2	63	62	60	445	38	169	22	276
	CYGB set 2	57	74	60	445	34	116	26	329
Hubers 2015	RASSF1A learning	42.5	96.5	73	86	31	3	42	83
	APC learning	52.1	70.9	73	86	38	25	35	61
	CYGB learning	56.2	74.4	73	86	41	22	32	64
	3OST2 learning	50.7	86	73	86	37	12	36	74
	PRDM14 learning	60.3	76.7	73	86	44	20	29	66
	FAM19A4 learning	86.3	29.1	73	86	63	61	10	25
	PHACTR3 learning	57.5	77.9	73	86	42	19	31	67
	RASSF1A validation	36.5	88.3	159	154	58	18	101	136
	APC validation	52.2	69.5	159	154	83	47	76	107
	CYGB validation	49.7	68.2	159	154	79	49	80	105
	3OST2 validation	49.7	85.1	159	154	79	23	80	131
	PRDM14 validation	64.8	74	159	154	103	40	56	114

	FAM19A4 validation	77.4	22.1	159	154	123	120	36	34
	PHACTR3 validation	60.4	62.3	159	154	96	58	63	96
Su 2016	RASSF1A training	45.3	86.2	117	174	53	24	64	150
	3OST2 training	49.3	84.5	117	174	58	27	59	147
	PRDM14 training	59.3	77.3	117	174	69	39	48	135
Hubers 2017	RASSF1A	17.2	92.6	56	217	10	16	46	201
	APC	58.6	56.6	56	217	33	94	23	123
	CYGB	51.7	45.9	56	217	29	117	27	100
	3OST2	10.3	86.1	56	217	6	30	50	187
	PRDM14	41.4	71.7	56	217	23	61	33	156
	FAM19A4	86.2	16.4	56	217	48	181	8	36
	PHACTR3	34.5	63.5	56	217	19	79	37	138
Hulbert 2017	SOX17	84	88	90	24	76	3	14	21
	TAC1	86	75	90	24	77	6	13	18
	HOXA7	63	92	90	24	57	2	33	22
	CDO1	78	67	90	24	70	8	20	16
	HOXA9	93	8	90	24	84	22	6	2
	ZFP42	87	63	90	24	78	9	12	15
Su 2018	HOXA9	80	51	127	159	102	78	25	81
	RASSF1A	29	96	127	159	37	6	90	153
	SOX17	85	89	127	159	108	17	19	142
	TAC1	87	76	127	159	110	38	17	121
Li 2021	3OST2	55	82	40	36	22	6	18	30
	APC	45	86	40	36	18	5	22	31
	DAPK	46	81	40	36	18	7	22	29
	FHIT	64	85	40	36	26	5	14	31
	GATA	67	58	40	36	27	15	13	21
	HOXA9	78	53	40	36	31	17	9	19

	MAGE	57	73	40	36	23	10	17	26
	p16	59	82	40	36	24	6	16	30
	PAX5	49	72	40	36	20	10	20	26
	DLC1	60	58	40	36	24	15	16	21
	PRDM14	66	75	40	36	26	9	14	27
	RASSF1A	57	75	40	36	23	9	17	27
	SOX17	85	70	40	36	34	11	6	25
	TAC1	89	76	40	36	36	9	4	27

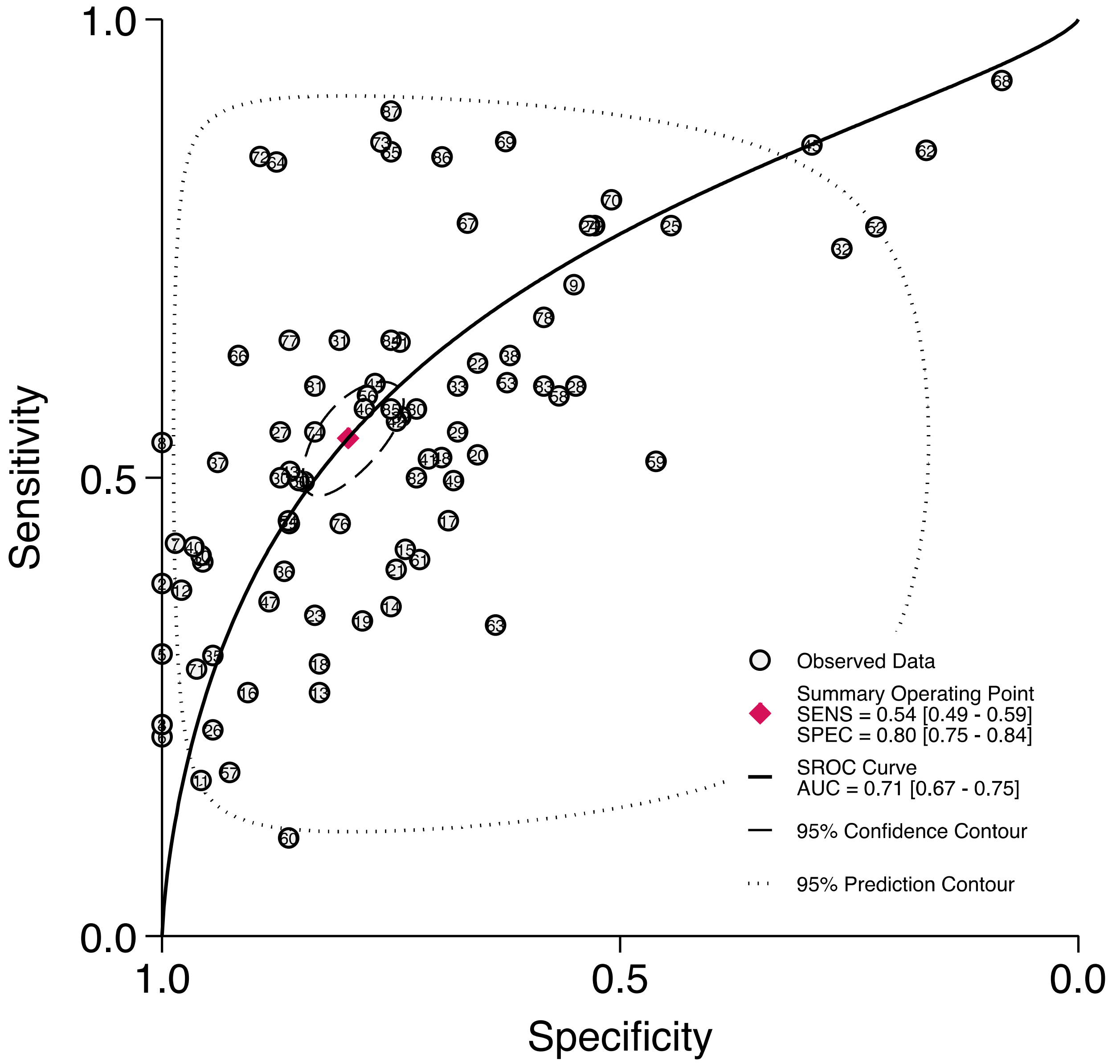

Figure S2. Hierarchical summary receiver operating characteristics plot. Each open circle represents a gene analyzed in an independent cohort and is identifiable with an ID. The summary point is represented by the red square, and the 95% confidence region and 95% prediction region are outlined in large dashes and small dashes, respectively.

Table S4. ID number, study ID, and gene name corresponding to Figure S2.

ID	Study ID	Gene name
1	Shivapurkar 2007	3OST2
2	Shivapurkar 2007	RASSF1A
3	Shivapurkar 2007	P16
4	Shivapurkar 2007	APC
5	Shivapurkar 2008 A	CYGB
6	vanderDrift 2008	RASSF1A supernatant
7	vanderDrift 2008	RASSF1A pellet
8	Shivapurkar 2008 B	TCF21
9	Hwang 2011	HOXA9
10	Hubers 2012	RASSF1A cumulative
11	Hubers 2012	APC cumulative
12	Hubers 2012	CYGB cumulative
13	Leng 2012	MGMT CO
14	Leng 2012	DAPK CO
15	Leng 2012	PAX5B CO
16	Leng 2012	Dal1 CO
17	Leng 2012	PCDH20 CO
18	Leng 2012	Jph3 CO
19	Leng 2012	Kif1a CO
20	Leng 2012	DAPK NM
21	Leng 2012	PAX5B NM
22	Leng 2012	PAX5A NM
23	Leng 2012	Dal1 NM
24	Leng 2012	GATA5 NM
25	Leng 2012	SULF2 NM
26	Leng 2012	CXCL14 NM
27	Hubers 2014 A	RASSF1A
28	Hubers 2014 A	APC
29	Hubers 2014 A	CYGB
30	Hubers 2014 A	3OST2
31	Hubers 2014 A	PRDM14
32	Hubers 2014 A	FAM19A4
33	Hubers 2014 A	PHACTR3
34	Hubers 2014 B	RASSF1A set 1
35	Hubers 2014 B	APC set 1
36	Hubers 2014 B	CYGB set 1
37	Hubers 2014 B	RASSF1A set 2
38	Hubers 2014 B	APC set 2

39	Hubers 2014 B	CYGB set 2
40	Hubers 2015	RASSF1A learning
41	Hubers 2015	APC learning
42	Hubers 2015	CYGB learning
43	Hubers 2015	3OST2 learning
44	Hubers 2015	PRDM14 learning
45	Hubers 2015	FAM19A4 learning
46	Hubers 2015	PHACTR3 learning
47	Hubers 2015	RASSF1A validation
48	Hubers 2015	APC validation
49	Hubers 2015	CYGB validation
50	Hubers 2015	3OST2 validation
51	Hubers 2015	PRDM14 validation
52	Hubers 2015	FAM19A4 validation
53	Hubers 2015	PHACTR3 validation
54	Su 2016	RASSF1A training
55	Su 2016	3OST2 training
56	Su 2016	PRDM14 training
57	Hubers 2017	RASSF1A
58	Hubers 2017	APC
59	Hubers 2017	CYGB
60	Hubers 2017	3OST2
61	Hubers 2017	PRDM14
62	Hubers 2017	FAM19A4
63	Hubers 2017	PHACTR3
64	Hulbert 2017	SOX17
65	Hulbert 2017	TAC1
66	Hulbert 2017	HOXA7
67	Hulbert 2017	CDO1
68	Hulbert 2017	HOXA9
69	Hulbert 2017	ZFP42
70	Su 2018	HOXA9
71	Su 2018	RASSF1A
72	Su 2018	SOX17
73	Su 2018	TAC1
74	Li 2021	3OST2
75	Li 2021	APC
76	Li 2021	DAPK
77	Li 2021	FHIT
78	Li 2021	GATA

79	Li 2021	HOXA9
80	Li 2021	MAGE
81	Li 2021	p16
82	Li 2021	PAX5
83	Li 2021	DLC1
84	Li 2021	PRDM14
85	Li 2021	RASSF1A
86	Li 2021	SOX17
87	Li 2021	TAC1

Table S5. Quality Assessment of Diagnostic Accuracy Studies 2, domain 1 – Patient selection.

Study ID	Was a consecutive or random sample of patients enrolled?	Supporting text	Was a case-control design avoided?	Did the study avoid inappropriate exclusions?	Supporting text	Could the selection of patients have introduced bias?	Is there concern that the included patients do not match the review question?	Supporting text
**Shivapurkar 2007**	No		No	Yes		Unclear risk	High risk	Controls included 4 patients with prior lung cancer.
**Shivapurkar 2008**	No		No	Yes		Unclear risk	Unclear risk	
**vanderDrift 2008**	No		No	Unclear		High risk	Low risk	
**Shivapurkar 2008**	No		No	Yes		Unclear risk	Low risk	
**Hwang 2011**	No		No	Yes		Unclear risk	Low risk	
**Hubers 2012**	Yes		Yes	Yes		Low risk	High risk	Including patients with recurrence and controls who were cancer-free for 3 years.
**Leng 2012**	No		No	Yes		Unclear risk	Low risk	
**Hubers 2014**	No		No	Unclear		High risk	High risk	Also included patients with lung cancer progression
**Hubers 2014**	Yes	"Two independent randomly selected study sets were composed from the prospectively	No	Yes		Low risk	Low risk	

		collected sputum bank"						
**Hubers 2015**	Unclear		No	Yes		Unclear risk	High risk	Because they included patients with disease recurrence and controls who were disease free for 3 years.
**Su 2016**	No		No	Yes		Unclear risk	Low risk	
**Hubers 2017**	No	99 controls were randomly selected, but the 56 cases were not and 120 other controls were not.	No	Unclear	The text mentions excluding 9 cases but not the reason for exclusion. There is a discrepancy between the number of controls mentioned in the text (219) and in Table 1 (217).	High risk	Unclear risk	Unclear because of the unclear exclusions.
**Hulbert 2017**	Unclear		Yes	Yes		Low risk	Low risk	
**Su 2018**	No		No	Yes		Unclear risk	Low risk	
**Li 2021**	Unclear		Yes	Yes		Low risk	Low risk	

Table S6. Quality Assessment of Diagnostic Accuracy Studies 2, domain 2 – Index test.

Study ID	Were the index test results interpreted without knowledge of the results of the reference standard?	If a threshold was used, was it pre-specified?	Could the conduct or interpretation of the index test have introduced bias?	Is there concern that the index test, its conduct, or interpretation differ from the review question?
**Shivapurkar 2007**	Yes	No	Unclear risk	Low risk
**Shivapurkar 2008**	Yes	Yes	Low risk	Low risk
**vanderDrift 2008**	Unclear	Unclear	Unclear risk	Unclear risk
**Shivapurkar 2008**	Yes	Yes	Low risk	Low risk
**Hwang 2011**	Unclear	Unclear	Unclear risk	Low risk
**Hubers 2012**	Unclear	Yes	Low risk	Low risk
**Leng 2012**	Yes	Unclear	Low risk	Low risk
**Hubers 2014**	Unclear	Yes	Low risk	Unclear risk
**Hubers 2014**	Yes	Yes	Low risk	Low risk
**Hubers 2015**	Yes	Yes	Low risk	Low risk
**Su 2016**	Unclear	No	Unclear risk	Low risk
**Hubers 2017**	Unclear	Yes	Low risk	Low risk
**Hulbert 2017**	Yes	Unclear	Low risk	Low risk
**Su 2018**	Unclear	No	Unclear risk	Low risk
**Li 2021**	Unclear	Yes	Low risk	Low risk

Table S7. Quality Assessment of Diagnostic Accuracy Studies 2, domain 3 – Reference standard.

Study ID	Is the reference standard likely to correctly classify the target condition?	Supporting text	Were the reference standard results interpreted without knowledge of the results of the index test?	Could the reference standard, its conduct, or its interpretation have introduced bias?	Is there concern that the target condition as defined by the reference standard does not match the review question?	Supporting text
**Shivapurkar 2007**	Yes		Yes	Low risk	Low risk	
**Shivapurkar 2008**	Unclear		Yes	Unclear risk	Low risk	
**vanderDrift 2008**	Yes		Yes	Low risk	Low risk	
**Shivapurkar 2008**	Unclear		Yes	Unclear risk	Low risk	
**Hwang 2011**	Yes		Yes	Low risk	Low risk	
**Hubers 2012**	Unclear		Yes	Unclear risk	Unclear risk	Reference standard not described.
**Leng 2012**	Yes		Yes	Low risk	Low risk	
**Hubers 2014**	Yes	Reference standard not specified.	Yes	Low risk	Low risk	
**Hubers 2014**	Unclear		Yes	Unclear risk	Unclear risk	Reference standard not described.
**Hubers 2015**	Yes		Yes	Low risk	Low risk	
**Su 2016**	Yes		Yes	Low risk	Low risk	
**Hubers 2017**	Yes		Yes	Low risk	Low risk	
**Hulbert 2017**	Yes		Yes	Low risk	Low risk	
**Su 2018**	Yes		Yes	Low risk	Low risk	
**Li 2021**	Yes		Yes	Low risk	Low risk	

Table S8. Quality Assessment of Diagnostic Accuracy Studies 2, domain 4 – Flow and timing.

Study ID	Was there an appropriate interval between index test(s) and reference standard?	Supporting text	Did all patients receive a reference standard?	Did patients receive the same reference standard?	Were all patients included in the analysis?	Supporting text	Could the patient flow have introduced bias?
**Shivapurkar 2007**	Unclear		Yes	Yes	Yes		Low risk
**Shivapurkar 2008**	Unclear		Unclear	Unclear	Yes		Unclear risk
**vanderDrift 2008**	Yes		Yes	Yes	No	Some were excluded due to low quality or level of DNA in the sample.	Low risk
**Shivapurkar 2008**	Unclear		Unclear	Unclear	Yes		Unclear risk
**Hwang 2011**	Unclear		Yes	Yes	Yes		Low risk
**Hubers 2012**	Unclear		Unclear	Unclear	Yes		Unclear risk
**Leng 2012**	No	Cohorts initiated in 1993, study published in 2012.	Yes	Yes	Yes		Low risk
**Hubers 2014**	Yes		Yes	Yes	Unclear		Low risk
**Hubers 2014**	No	Samples were collected in 2000-2005, the follow-up data in 2006, and the paper was published in 2014.	Unclear	Unclear	Yes		Unclear risk
**Hubers 2015**	Yes		Yes	Yes	No		Low risk
**Su 2016**	Unclear		Yes	Yes	Yes		Low risk
**Hubers 2017**	No	Samples collected around 2005, but the study was published in 2017. Storage presumably around 10 years.	Yes	Yes	No		Unclear risk
**Hulbert 2017**	No	Study initiated in 2007, this paper was published in 2017.	Yes	Yes	Yes		Low risk
**Su 2018**	Unclear		Yes	Yes	Yes		Low risk
**Li 2021**	Unclear		Yes	Yes	Yes		Low risk

Table S9. Funding and conflicts of interest.

Study ID	Funding: How was the study funded?	Conflicts of interest
**Shivapurkar 2007**	Public/non-profit funding sources	Not reported
**Shivapurkar 2008**	Public/non-profit funding sources	None
**vanderDrift 2008**	Public/non-profit funding sources	Not reported
**Shivapurkar 2008**	Public/non-profit funding sources	Not reported
**Hwang 2011**	Public/non-profit funding sources	None
**Hubers 2012**	Public/non-profit funding sources	None
**Leng 2012**	Company/corporate funding sources	Yes, pertaining to the funding sources
**Hubers 2014**	Public/non-profit funding sources	None
**Hubers 2014**	Public/non-profit funding sources	None
**Hubers 2015**	Public/non-profit funding sources	Yes, but not pertaining to the funding sources
**Su 2016**	Public/non-profit funding sources	None
**Hubers 2017**	Mix of corporate and public	Yes, but not pertaining to the funding sources
**Hulbert 2017**	Public/non-profit funding sources	Yes, but not pertaining to the funding sources
**Su 2018**	Public/non-profit funding sources	None
**Li 2021**	Public/non-profit funding sources	None

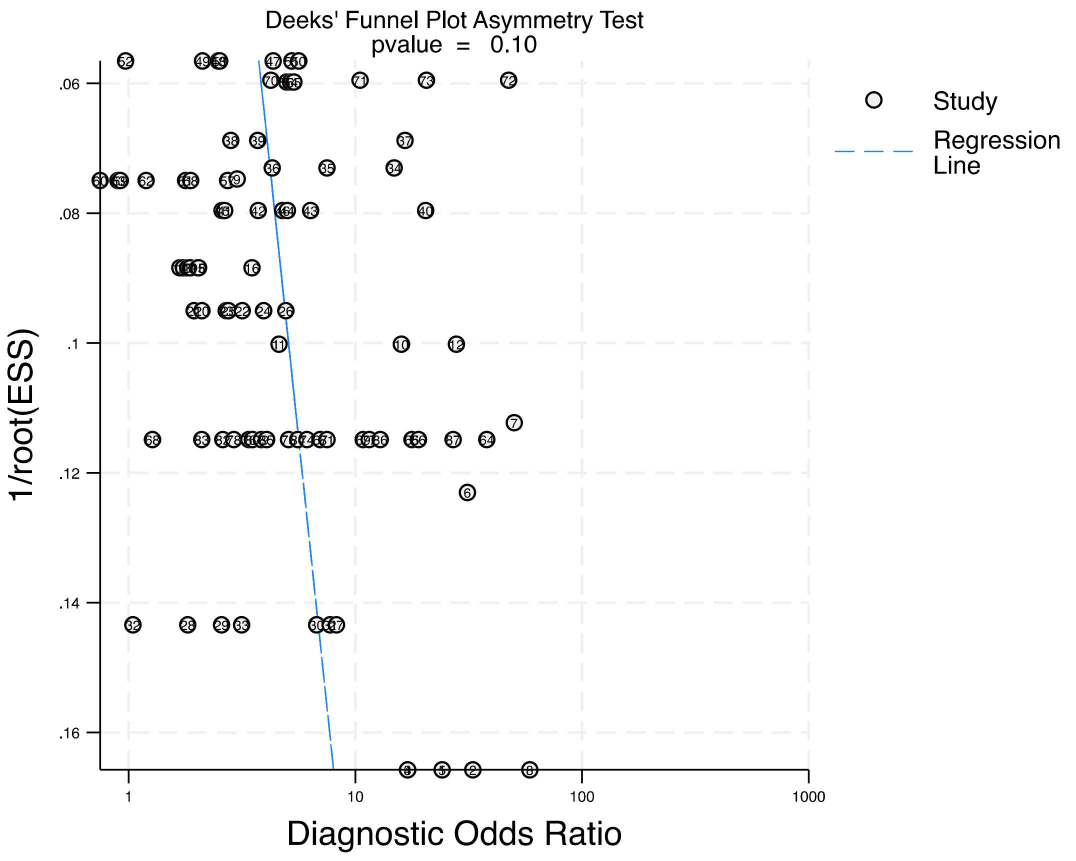

Figure S3. Deek's Funnel Plot. The diagnostic odds ratio is plotted against the $1/ESS^{1/2}$, where ESS is the effective sample size. Each circle represents a gene analyzed in an independent cohort, and the regression result is illustrated by a blue dashed line.
